# Editorial: Understanding the socio-emotional and socio-cognitive developmental pathways in children with sensory impairment

**DOI:** 10.3389/fpsyg.2022.1118451

**Published:** 2022-12-22

**Authors:** Livio Provenzi, Monica Gori, Laura Maffongelli, Sabrina Signorini

**Affiliations:** ^1^Department of Brain and Behavioral Sciences, University of Pavia, Pavia, Italy; ^2^Developmental Psychobiology Lab, IRCCS Mondino Foundation, Pavia, Italy; ^3^Unit of Visually Impaired People, Italian Institute of Technology (IIT), Genoa, Italy; ^4^Department of Psychology, Johannes-Gutenberg University, Mainz, Germany; ^5^Developmental Neuro-Ophthalmology Unit, IRCCS Mondino Foundation, Pavia, Italy

**Keywords:** sensory processing, sensory deficit, children, parenting, autism, preterm (birth), neuroimaging

Humans—both children and adults—crave to make meaning of reality (Tronick, 2005). Developing cohesive representations and reliable expectations of the physical and social world in which we move is key to achieving optimal developmental outcomes, health, and well-being (Rochat, 2003; Geangu et al., 2011). As we move on to create meaning, we also obtain, develop, and refine specific skills that we use to cope, engage, and transact with the social and physical environment. Socio-emotional and socio-cognitive skills are critically important to reach this goal, as they provide infants' with critical information bits that favor self-other differentiation and the emergence of consistent representations of self-other interactive exchanges (Rochat and Striano, 2002). Not surprisingly, these processes of meaning-making and socio-emotional/socio-cognitive development in humans occur and envelop within the early interactions between children and their caregivers, adult conspecifics that take care of them (Tronick and Beeghly, 2011).

The socio-emotional skills provide the developing child with the ability to maintain an optimal regulation of inner states and to adjust them to fit with the ongoing changes occurring in the surrounding environment. Emotion regulation (Cole et al., 2004), secure attachment (De Carli et al., 2016), and empathy (Vaish and Warneken, 2012) are only specific terms that we give to as many key outcomes of child socio-emotional development. Yet, achieving adaptive emotion regulation skills (Jazaieri et al., 2013), developing a feeling of security in the relationship with the caregivers (Cyr et al., 2010), and is capable of empathically resonating with a conspecific (Psychogiou et al., 2008) are universally considered as powerful protective factors for psychological health later in life.

The socio-cognitive skills instead represent a peculiar form of human intelligence that allows us to access special cognitive representations of the world when we cope with social challenges. Socio-cognitive skills include the capacity to represent in our mind the mind of other conspecifics (Leslie et al., 2004), the confidence we have about our ability to influence the mind of others (Salvadori et al., 2015), the possibility to cooperate to achieve collaborative goals in competitive and challenging conditions (Tomasello, 2020). Concerning socio-emotional skills, these special abilities also create the conditions for optimal psychological development and adaptive life outcomes in adult life.

When we refer to the inherent need to make meaning of reality, we sometimes use the expression “making sense” of the world. Something makes sense for us when we identify specific causes and/or consequences of an event, an action, or a phenomenon that we can describe and share with others. Of course, there is no doubt that we rely heavily on our senses to make meaning of reality and that we do so from the beginning of our lives; our first emergent sense of self is a bodily or nuclear self that is cohesive as much as we can integrate the information that we obtain from different sensory sources into an integrated view of ourselves, of others, of the world. Yet, what happens when these fundamental sources of primary information on life are challenged, impeded, deficient or broken? This question inspired us to design the present Research Topic and to encourage researchers and clinicians to provide scientifically valuable stories about how humans make sense of the world under altered sensory conditions. The call for papers resulted in seven papers seeking specific responses to this question from different perspectives.

Narayan et al. investigated the cerebellum's role beyond normal sensory processing, assessing cerebellar alterations in children with sensory processing dysfunction (SPD). They reported reduced microstructural integrity in the cerebellum of individuals with SPD, especially in the superior and middle cerebellar peduncles. They also reported on minor microstructural integrity of cerebral peduncles in the same subjects compared to typically developing controls, which might link to abnormal tactile and auditory behaviors. These findings are critical to inform early diagnostic approaches and treatments for children with SPD.

The group of Teresa Farroni (Valori et al.) has targeted the sensorimotor processing alterations that characterize many individuals with autism spectrum disorders (ASD). The authors have highlighted how innovative technologies—such as head-mounted displays in the context of immersive virtual reality—might be used to create explorative environments to improve the perceptual and motor experience of the world in children with ASD. By highlighting the challenges and the benefits of virtual vs. natural environments for the sensorimotor profile of children with ASD, Valori et al. provide valuable insights for clinicians and researchers to further integrate technologies into beneficial tools for more innovative functional diagnosis and treatment.

Using event-related potentials (ERPs), Sun et al. evaluate the neural processing of different emotional prosodies in newborns of diabetic mothers. In addition, they assessed the presence of ASD risk at 24 months using the Modified Checklist for Autism in toddlers (M-CHAT). Their findings suggest that an early alteration in the electrophysiological processing of prosodic stimuli—especially a lower mismatch response in the frontal lobe during fearful prosodies—was significantly associated with higher scores at the M-CHAT.

As applying research findings and data-driven models to specific therapeutic and rehabilitation settings is never an immediate process, disposing of flexible and straightforward evidence-based tools for behavioral and sensory profile evaluation is a crucial resource for clinicians. Chen et al. contributed to the field by publishing a simplified model of learning accomplishment profiling (C-LAP), demonstrating appropriate sensitivity in training and validation in a community of Chinese toddlers between 24 and 36 months.

Preterm infants are exposed to a sensory environment they are unprepared to cope with. Intense lights and sounds and invasive and painful procedures constitute—in the partial absence of caregivers' neuroprotection and closeness—a well-known source of developmental risk that might alter the neurobiological pathways of preterm infants' sensory processing. Zheng et al. test the efficacy of an early sensory stimulation program (5-ISS) to improve preterm infants' socio-emotional development. In this randomized controlled trial, they successfully documented that providing sensory neuroprotection to preterm infants during the intensive care unit stay can improve socio-emotional and stress regulation outcomes.

Ludwig and Welch contribute wisely to the article collection by providing a theoretical discussion of the socio-emotional and socio-cognitive implications of the polyvagal regulation of the parasympathetic system in mammals and humans, especially—in both the evolutionary and ontogenetic frameworks. By updating previous theoretical propositions and confronting them with the most recent data from scientific investigations, they build the rationale by which early parent-infant socio-emotional exchange would be craven my mutual autonomic state plasticity. According to this model, the authors also provide insights for enhancing our approaches to treating mother-infant dyads in atypical and risky conditions.

Finally, focusing on the contingent condition of the COVID-19 pandemic, Gori et al. explored how blind individuals subjectively experience the restrictions and isolation that came together with the mitigation strategies adopted to cope with this unprecedented healthcare emergency. In their study, blind individuals had similar challenges to sighted controls in adapting to the modified life conditions; nonetheless, the source of more incredible frustration and stress for blind subjects were practical and logistical issues (e.g., loss of personal autonomy, self-protection measures, use of public transport). These findings highlight the need to develop tailored responses to the condition of the most fragile individuals when adopting country-level responses to large-scale emergencies.

The picture we obtained from this Research Topic needs to be more conclusive. Yet, this article collection might illuminate specific open questions and directions of future investigations that will contribute to our understanding of the mechanisms by which—in typical and atypical developing conditions—we make sense of reality by relying on our senses.

## Author contributions

LP drafted the initial version of the manuscript. All authors contributed critically and agreed upon final submission.
